# Geriatric assessment in undergraduate geriatric education – a structured interpretation guide improves the quantity and accuracy of the results: a cohort comparison

**DOI:** 10.1186/1472-6920-13-116

**Published:** 2013-08-30

**Authors:** Elisabeth Igenbergs, Tobias Deutsch, Thomas Frese, Hagen Sandholzer

**Affiliations:** 1 Department of Primary Care, Leipzig Medical School, Philipp-Rosenthal-Straße 55, Leipzig 04103, Germany

**Keywords:** Comprehensive geriatric assessment, Geriatric education, General practice, Undergraduate medical education, Interpretation guide

## Abstract

**Background:**

With regard to the growing proportion of elderly multimorbid patients, a sound undergraduate geriatric education becomes more important. Therefore we included the execution and interpretation of a comprehensive geriatric assessment (CGA) into a mandatory two-week clerkship at a general practitioner's office. The present study examined the effect of a guide structuring the students’ considerations when interpreting a CGA on the quantity and accuracy of the documented findings and conclusions.

**Methods:**

We compared random samples out of two cohorts of fourth-year medical students (interpreting the CGA with and without using the structured interpretation guide) completing their clerkship between April 2006 and March 2008 with regard to the content of the CGA interpretation and the grades the students achieved for their clerkship documentation, which were substantially determined by the quantity and accuracy of the documentation and interpretation of the CGA.

**Results:**

The structured interpretation guide led to significantly more mentions of aspects that have to be considered in geriatric patient care and to a higher documentation rate of respective positive results. Furthermore, students who analysed the CGA by using the interpretation guide achieved significantly better grades.

**Conclusions:**

An additional tool structuring medical students’ considerations when interpreting a CGA increases the quantity and the accuracy of the documented findings and conclusions. This may enhance the students’ learning gain.

## Background

During the past two decades, the life expectancy of elderly people has increased in all European countries [1]. Several studies suggest an expansion of the time people will live with multiple diseases and disability at the end of their life [2,3]. Conclusively, the treatment of elderly multimorbid patients will make up a larger part of the clinical work of nearly every medical specialty [4,5]. Furthermore, a greater number of trained geriatricians will be needed [5]. Already today there is a shortage of young physicians who decide to take up a career in that area [5,6]. Thus, it is important to motivate undergraduate medical students to explore the field of geriatrics [6]. A well-thought-out implementation of the field into the undergraduate curriculum seems essential [4-7]. Tullo et al. demonstrated in a current literature review (2010) that a wide range of undergraduate educational interventions in geriatrics potentially improve the respective knowledge, skills and attitudes [7].

We established a mandatory office-based two-week general practice clerkship for all fourth-year medical students. An (in-home) comprehensive geriatric assessment (CGA) was implemented as one core component of the clerkship. Because CGAs are designed to cover a broad spectrum of elderly patients’ concerns in different areas of life, they seem particularly suitable for sensitizing medical students to the complexity of the field. Furthermore, it has been shown that CGAs are valuable and successful tools for the comprehensive diagnostic and therapy of elderly patients [8-11].

Initial experience revealed substantial difficulties of the students to handle the amount and complexity of the results of the CGA. Thus, we developed a guide serving as a structuring aid for non-geriatricians when documenting and interpreting the results of the CGA.

The present study was set out to examine whether this structured interpretation guide increases the quantity and accuracy of the documented findings and conclusions that students draw from a CGA.

## Methods

### Sampling and design

We analysed the data of fourth-year (of a six-year curriculum) medical students who completed their mandatory two-week general practice clerkship between April 2006 and March 2008 in offices of general practitioners collaborating with the Department of Primary Care of the Leipzig Medical School (general practitioner teachers for undergraduates [12]). The clerkship with one-to-one tutoring included a home visit to an elderly patient to execute and interpret a CGA. In 2007, a specially developed structured interpretation guide (“checklist”) was implemented as an aid for documenting and interpreting the results of the CGA. In the present study, random samples of students of respectively two years before and after the implementation of the interpretation guide were compared. These cohorts are in the following referred to as “non-checklist-group” (Non-CL-group) and “checklist-group” (CL-group). Both groups were asked for a documentation and interpretation of the findings from the CGA. While the Non-CL-group used a few very general open questions with free-text answers the CL-group was instructed to use the checklist.

Random sampling was conducted on the basis of the alphabetical order of surnames in the semester list. About 50 students with surnames from A onwards were chosen from each semester, resulting in a sample of n = 206 (Non-CL-group: n = 100; CL-group: n = 106) out of a total of n = 706 medical students.

Comparisons between the two cohorts were made regarding the frequencies with which the students mentioned the content specified by the items of the structured interpretation guide in general (item content addressed) and the frequencies of documented positive results (results that should be considered in the medical care process) regarding the respective item content. Furthermore, we compared the grades the students achieved for the documentation of their two-week general practice clerkship. These grades were substantially determined by the quantity and accuracy of the documentation and interpretation of the CGA as evaluated by trained employees of the Department of Primary Care following a standardized scheme. Grades ranged thereby from “1” (excellent) to “5” (fail).

Structural comparability between the two cohorts with regard to the students and the examined patients was required as a prerequisite to interpret an effect of the structured interpretation guide. Therefore, we compared the two student cohorts regarding age and gender. Appropriate geriatric patients were selected by their attending general practitioner. The patients were compared with regard to age, gender, housing situation, civil status, and number of children as objective data, as well as the degree of morbidity. As indicators for the degree of morbidity, we used patients’ self-assessments within two items of the COOP/WONCA charts (“physical fitness” and “daily activities”) [13], the results of short-screenings for depression and dementia, the body mass index, the number of drugs taken, and the number of diagnoses documented by the students at the end of the CGA.

### Comprehensive geriatric assessment

The geriatric assessment tool used in this study was the STEP (“Standardised Assessment for Elderly People in Primary Care”), described in detail by Sandholzer and colleagues [14]. At Leipzig Medical School, the STEP was augmented by the Mini-Mental State Examination (MMSE), two items of the COOP/WONCA charts (an established screening instrument that measures functional capacity at a given time), and the Barthel Index [13,15,16]. The STEP is an established comprehensive geriatric assessment tool for use in primary care addressing the relevant topics of elderly patients [11,14,17].

### Structured interpretation guide

The interpretation guide structuring the students’ interpretation of the CGA results was modified based on the experiences and feedback of the students who used the initial form. The initial form was extensively oriented to the interpretation guideline for systematic geriatric assessments as described by Mayer & Sandholzer [18]. The interpretation guide is especially attuned to the STEP and scans through the following topics: medication, physical fitness and mobility, general and domestic care, (instrumental) activities of daily living (ADL/IADL), social environment, common geriatric cardinal symptoms, physical status (medical history and examination), mood and cognition, lifestyle, vaccination status, hospital admissions, and noticeable problems in the course of the actual physical examination. The detailed content of the structured interpretation guide is presented in an additional file (see Additional file 1). With regard to every aspect, the students are asked to think about further diagnostics, counselling, treatment, and referral, if necessary.

### Statistical analysis

The anonymised data analysis was performed with SPSS 15.0®. Apart from descriptive statistics, frequency and mean score comparisons were performed to reveal significant differences between the two cohorts. For frequency comparisons, we used the Chi^2^-Test. Mean scores were compared by using the independent samples *t*-test and the Mann–Whitney *U*-test depending on the presence of normal distribution according to a Kolmogorov-Smirnov-Test. Statistical significant differences were assumed at an error rate of ≤ 5% (p < 0.05).

### Ethical approval

Our investigation was in accordance to the Declaration of Helsinki. According to the regulations of the ethics committee of the Leipzig Medical School an explicit ethical approval was deemed not necessary. All patients consented to the anonymous use of their data for accompanying scientific research with regard to medical education.

## Results

### Sample characteristics and comparability of the cohorts

Overall, 58.7% (n = 121) of the students were female. The average age was 24.5 ± 2.7 years (range from 21 to 38 years). There were no significant differences between the Non-CL-group and the CL-group regarding the students’ gender distribution (60.0% vs. 57.5% females; *χ*^2^ = 0.128, p = 0.721) and age (24.5 ± 2.8 vs. 24.4 ± 2.6 years; p = 0.864). The comparison between the two cohorts with regard to the patients’ characteristics is presented in Table 1. There were no significant differences, except for the number of diagnoses documented by the students.

**Table 1:** Patient sample characteristics - cohort comparison

**Characteristic**	Frequency (percent) *		**p**
	**Non-CL-group**	**CL-group**	
Female	75.0	73.6	0.816
Age (years; mean ± SD)	81.3 ± 8.9	80.9 ± 7.6	0.715
Civil status			
living alone	16.2	18.4	
married	26.3	25.2	
divorced	9.1	11.7	
separated	1.0	0.0	
widowed	47.5	44.7	0.806
Housing situation			
at the family’s/children’s place	9.0	8.7	
nursing home	22.0	19.4	
own apartment	69.0	71.8	0.893
WONCA/COOP chart “daily activities”			
During the past two weeks … difficulty			
doing usual activities or tasks			
no difficulty at all	12.0	7.7	
a little bit of difficulty	13.0	12.5	
some difficulty	32.0	26.9	
much difficulty	36.0	38.5	
could not do	7.0	14.4	0.397
WONCA/COOP chart “physical fitness”	…		
During the past 2 weeks … hardest	…		
physical activity for at least two	…		
minutes	…		
very heavy (e.g. fast running)	3.0	3.8	
heavy (e.g. jogging)	1.0	4.8	
moderate (e.g. fast walking)	16.2	12.4	
light (e.g. normal walking)	19.2	21.0	
very light (e.g. slow walking)	49.5	50.5	
not able	11.1	7.6	0.606
Depression screening positive	34.3	43.8	0.166
Dementia screening positive	16.7	10.6	0.208
BMI (mean ± SD)	27.6 ± 6.0	28.1 ± 6.1	0.625
Number of drugs taken (mean ± SD)	6.0 ± 2.5	6.6 ± 3.2	0.233
Number of diagnoses (mean ± SD)	6.1 ± 2.2	7.0 ± 2.5	0.009

* Unless otherwise indicated; n varies slightly due to missing values.

### Interpretation of the comprehensive geriatric assessment

With regard to every topic addressed within the structured interpretation guide, the frequencies of mentions of the content specified by the respective items (item content addressed) were significantly higher in the CL-group. For 11 of 12 topics the frequencies of documented positive results regarding the respective item content was significantly higher in the CL-group. An overview on all topics and the corresponding frequencies for the two groups is given in Table 2.

**Table 2:** **Frequencies of mentions of the content specified by the items of the structured interpretation guide (item content addressed) and frequencies of documented positive results regarding the respective item content - cohort comparison** *

**Topic**	**Item content addressed**			**Positive results**		
	**Non-CL-group (%)**	**CL-group (%)**	**p**	**Non-CL-group (%)**	**CL-group (%)**	**p**
Medication	5.7	97.4	< 0.001	4.0	24.9	< 0.001
Physical fitness and mobility	19.0	98.1	0.001	17.5	82.5	< 0.001
General and domestic care	8.4	92.8	< 0.001	6.0	35.8	< 0.001
(instr.) activities of daily living (ADL/IADL)	1.0	90.6	< 0.001	1.0	38.5	< 0.001
Social environment	3.7	99.3	< 0.001	1.2	26.4	< 0.001
Common geriatric cardinal symptoms	7.0	99.6	< 0.001	7.0	51.9	< 0.001
Physical status (medical history and examination)	5.2	96.2	< 0.001	5.2	34.0	< 0.001
Mood and cognition	53.3	98.1	< 0.001	15.7	21.7	0.055
Lifestyle	2.7	100.0	< 0.001	2.7	18.9	< 0.001
Vaccination status	1.3	98.7	< 0.001	1.3	18.2	< 0.001
Hospital admissions	0.0	100.0	< 0.001	0.0	62.3	< 0.001
Physical examination	4.0	98.1	< 0.001	3.5	29.2	< 0.001

* The presented relative frequencies describe the proportion of mentions/positive results relative to the maximum number of possible mentions/positive results for each topic (number of items per topic multiplied by group size).

### Grades

Table 3 shows the results of the cohort comparison concerning the frequency distributions of the grades the students achieved for their clerkship documentation. The students of the CL-group received nearly three times more often the best grade “excellent”. The CL-group achieved a significantly better grade point average than the Non-CL-group (mean ± SD: 1.3 ± 0.6 vs. 2 ± 0.9; p < 0.001).

**Table 3:** Frequency distributions of the grades the students achieved for their clerkship documentation – cohort comparison

	**Frequencies of the achieved grades (%)**					***χ***^**2**^	**p**
	**1 “excellent”**	**2 “good”**	**3 “satisfactory”**	**4 “pass”**	**5 “fail”**		
CL-group	74.5	18.9	6.6	0.0	0.0	45.4	< 0.001
Non-CL-group	29.0	53.0	12.0	3.0	3.0		

## Discussion

The present study examined the effect of a guide structuring the students’ considerations when interpreting a CGA on the quantity and accuracy of the documented findings and conclusions. In our sample, the structured interpretation guide led to substantially more mentions of aspects that have to be considered in geriatric patient care and to a higher documentation rate of respective positive results. Furthermore, students who analysed the CGA by using the interpretation guide achieved better grades.

With regard to the students’ age and gender distribution there were no relevant differences between our sample and the total of medical students who completed the mandatory clerkship at the Leipzig Medical School in the respective years, indicating representativeness. The student cohorts were comparable concerning age and gender. The patient groups examined by the two student cohorts were comparable regarding the characteristics presented in Table 1. The only exception was a significant difference in the number of diagnoses between the two patient cohorts with an average of one diagnosis more in the group of patients assessed by the student cohort using the structured interpretation guide. As the number of diagnoses was collected from the students’ written interpretation after the execution of the CGA, this discrepancy is likely to be due to the use of the interpretation guide. Given the comparability of the two patient groups with regard to all other variables, they can nevertheless be considered as structurally comparable.

The structured interpretation guide led to a substantial increase with regard to the mention of aspects that have to be considered in geriatric patient care by the students (Table 2). Although the task for both student groups was a detailed interpretation of the CGA findings, the structured interpretation guide presumably encouraged students to address all relevant considerations, while those without this guide possibly did not mention aspects without positive results. The fact that we also found significant differences regarding the frequency of positive results suggests that the structuring interpretation guide led to a more accurate analysis and documentation of the CGA findings. Substantial rates of under-documentation by medical students were already shown by Szauter et al. and Worzala et al. for the context of post-encounter notes, possibly due to forgetfulness or inaccurate decisions on what is relevant and what is not [19,20]. In accordance with the results of the present study, Deering et al. showed that the use of a standard checklist as a documentation aid results in significantly improved delivery notes for shoulder dystocia [21].

In our sample, students using the structuring interpretation guide achieved substantially better grades for the documentation of their clerkship (Table 3). Considering the fact that these grades were substantially determined by the quantity and accuracy of the documentation and interpretation of the CGA, this indicates that the students’ analysis of the CGA not only improved in quantitative terms, but was also accurate with regard to content.

The use of the interpretation guide structuring the students’ considerations when interpreting a CGA in the present study may have encouraged medical students’ exploration of the multiple dimensions addressed by the CGA. This might have enhanced their learning gain regarding the field of geriatrics. In their review about the educational benefit of portfolios, which could be very broadly defined as a kind of “checklist”, Buckley et al. found evidence for improved knowledge and understanding as well as increased self-awareness and engagement in reflection on the part of the students [22]. There is still a need for further research concerning the general benefit of the integration of home visits to elderly patients including a CGA within undergraduate medical education. Previous studies have shown promising results. For instance, Medina-Walpole et al. found that respective experiences successfully enhanced the geriatric curricular content and positively affected students’ attitudes towards chronically ill and homebound elderly persons [23]. Furthermore, Denton et al. described a positive effect, especially with regard to attitudes [24]. Our results indicate that undergraduate medical students need a structuring tool serving as interpretation aid to handle the complexity of a CGA. The structured and intensified analysis might enhance the learning gain with regard to the field of geriatrics. In a broader context, our results raise questions concerning the benefit of a structured interpretation guide for CGAs not only for undergraduate students, but also for practising (family) physicians without special geriatric training. Further research in this area seems desirable.

### Strengths and limitations

To the best of our knowledge, this study is internationally the first to examine the effect of a structured interpretation guide on the quantity and accuracy of the documented findings and conclusions medical students draw from a CGA. Moreover, the implementation of mandatory CGA for all undergraduate medical students is an innovation itself. The finding of an average of one diagnosis more within the group of patients assessed by the student cohort using the structured interpretation guide may be argued. As already mentioned, this discrepancy is likely to be caused by the use of the structured interpretation guide itself and not by a limited structural comparability of the patient cohorts. It also seems unlikely that the large extent of the group-differences reported in the results can be explained solely by that aspect. A second limitation could be that the patient data we compared to demonstrate structural comparability between the two cohorts were collected by the students in the course of the CGA and were not verified independently. However, most of the data were objective or independent from the students’ competencies (age, gender, housing situation, civil status, number of children, standardised self-assessments based on the two items of the COOP/WONCA charts, standardised short-screenings for depression and dementia, BMI). Only the assessment of data regarding the number of drugs taken and the number of diagnoses may depend on the students’ competencies. Finally, the fact that the compared student cohorts studied in different years could be discussed. Possible differences, particularly with regard to the curriculum previous to the mandatory two-week general practice clerkship, might have influenced the students’ performance regarding the CGA interpretation. However, to our knowledge there were no respective changes in the (geriatric) curriculum between 2006 and 2008.

## Conclusions

An additional interpretation guide structuring medical students’ considerations when interpreting a CGA increases the quantity and the accuracy of the documented findings and conclusions. This may enhance the students’ learning gain for the complex field of geriatrics and medical care for elderly outpatients. With regard to the growing number of elderly multimorbid patients, a sound teaching of geriatrics in undergraduate medical education is essential. Further research should elucidate the students’ evaluation regarding the additional interpretation guide and whether such a tool may also improve general practitioners’ interpretation of a CGA.

## Competing interests

The authors declare that they have no competing interests.

## Authors’ contributions

EI wrote the first draft of the paper, conducted the data acquisition, and contributed to the statistical analysis and the interpretation of the results. TD helped to write the paper and contributed to the analysis and the interpretation of the results. TF helped to interpret the results and revised the manuscript. HS conceived of the study, contributed to its conception and design and revised the manuscript. All authors approved the final version of the manuscript submitted for publication.

## Pre-publication history

The pre-publication history for this paper can be accessed here:

http://www.biomedcentral.com/1472-6920/13/116/prepub

## Supplementary Material

Additional file 1 Topics and content of the structured interpretation guide – an overview. Click here for file

## References

[B1] Eurostat Mortality and life expectancy statistics 2011 http://epp.eurostat.ec.europa.eu/statistics_explained/index.php/Mortality_and_life_expectancy_statistics

[B2] Braunseis F Deutsch T Frese T Sandholzer H The risk for nursing home admission (NHA) did not change in ten years - a prospective cohort study with five-year follow-up Arch Gerontol Geriatr 2012 54 e63 e67 10.1016/j.archger.2011.06.023 21871672

[B3] Crimmins EM Beltrán-Sánchez H Mortality and morbidity trends: is there compression of morbidity? J Gerontol B Psychol Sci Soc Sci 2011 66 75 86 21135070 10.1093/geronb/gbq088 PMC3001754

[B4] Goldenhar LM Margolin EG Warshaw G Effect of extracurricular geriatric medicine training: a model based on student reflections on healthcare delivery to elderly people J Am Geriatr Soc 2008 56 548 552 10.1111/j.1532-5415.2007.01554.x 18179485

[B5] Weiss BD Fain MJ Geriatric education for the physicians of tomorrow Arch Gerontol Geriatr 2009 49 Suppl 2 S17 S20 20005420 10.1016/S0167-4943(09)70007-X

[B6] Adelman RD Capello CF LoFaso V Greene MG Konopasek L Marzuk PM Introduction to the older patient: a "first exposure" to geriatrics for medical students J Am Geriatr Soc 2007 55 1445 1450 10.1111/j.1532-5415.2007.01301.x 17767689

[B7] Tullo ES Spencer J Allan L Systematic review: helping the young to understand the old. Teaching interventions in geriatrics to improve the knowledge, skills, and attitudes of undergraduate medical students J Am Geriatr Soc 2010 58 1987 1993 10.1111/j.1532-5415.2010.03072.x 20840458

[B8] Huss A Stuck AE Rubenstein LZ Egger M Clough-Gorr KM Multidimensional preventive home visit programs for community-dwelling older adults: a systematic review and meta-analysis of randomized controlled trials J Gerontol A Biol Sci Med Sci 2008 63 298 307 10.1093/gerona/63.3.298 18375879

[B9] Frese T Deutsch T Keyser M Sandholzer H In-home preventive comprehensive geriatric assessment (CGA) reduces mortality-A randomized controlled trial Arch Gerontol Geriatr 2012 55 639 644 10.1016/j.archger.2012.06.012 22790107

[B10] Stuck AE Egger M Hammer A Minder CE Beck JC Home visits to prevent nursing home admission and functional decline in elderly people: systematic review and meta-regression analysis JAMA 2002 287 1022 1028 10.1001/jama.287.8.1022 11866651

[B11] Piccoliori G Gerolimon E Abholz HH Geriatric assessment in general practice using a screening instrument: is it worth the effort? Results of a South Tyrol Study Age Ageing 2008 37 647 652 10.1093/ageing/afn161 18703519

[B12] Lippmann S Frese T Herrmann K Scheller K Sandholzer H Primary care research - trade-off between representativeness and response rate of GP teachers for undergraduates Swiss Med Wkly 2012 142 w13537 22430810 10.4414/smw.2012.13537

[B13] van Weel C König-Zahn C Touw–Otten FWMM van Duijn NP Meyboom-de Jong B Measuring functional health status with the COOP/WONCA Charts: a manual 1995 Groningen: Northern Centre of Health Care Research (NCH)

[B14] Sandholzer H Hellenbrand W Renteln-Kruse W Van Weel C Walker P [STEP - standardized assessment of elderly people in primary care] Dtsch Med Wochenschr 2004 129 Suppl 4 S183 S226 15592957 10.1055/s-2004-836107

[B15] Folstein MF Folstein SE McHugh PR "Mini-mental state". A practical method for grading the cognitive state of patients for the clinician J Psychiatr Res 1975 12 189 198 10.1016/0022-3956(75)90026-6 1202204

[B16] Mahoney FI Barthel DW Functional Evaluation: The Barthel Index Md State Med J 1965 14 61 65 14258950

[B17] Frese T Franke M Keyser M Rurik I Sandholzer H Primary care guidelines for geriatric assessment: a structured, comparative analysis Z Gerontol Geriatr 2012 45 224 229 10.1007/s00391-011-0219-9 21805190

[B18] Mayer K Sandholzer H Sandholzer H Das geriatrische Assessment Allgemeinmedizin 2006 4 Aachen: Shaker Verlag 385 393

[B19] Szauter KM Ainsworth MA Holden MD Mercado AC Do students do what they write and write what they do? The match between the patient encounter and patient note Acad Med 2006 81 Suppl S44 S47 10.1097/00001888-200610001-00012 17001133

[B20] Worzala K Rattner SL Boulet JR Majdan JF Berg DD Robeson M Veloski JJ Evaluation of the congruence between students' post-encounter notes and standardized patients' checklists in a clinical skills examination Teach Learn Med 2008 20 31 36 10.1080/10401330701798253 18444183

[B21] Deering SH Tobler K Cypher R Improvement in documentation using an electronic checklist for shoulder dystocia deliveries Obstet Gynecol 2010 116 63 66 10.1097/AOG.0b013e3181e42220 20567169

[B22] Buckley S Coleman J Davison I Khan KS Zamora J Malick S Morley D Pollard D Ashcroft T Popovic C Sayers J The educational effects of portfolios on undergraduate student learning: a Best Evidence Medical Education (BEME) systematic review. BEME Guide No. 11 Med Teach 2009 31 282 298 10.1080/01421590902889897 19404891

[B23] Medina-Walpole A Heppard B Clark NS Markakis K Tripler S Quill T Mi Casa o Su Casa? Assessing function and values in the home J Am Geriatr Soc 2005 53 336 342 10.1111/j.1532-5415.2005.53124.x 15673362

[B24] Denton GD Rodriguez R Hemmer PA Harder J Short P Hanson JL A prospective controlled trial of the influence of a geriatrics home visit program on medical student knowledge, skills, and attitudes towards care of the elderly J Gen Intern Med 2009 24 599 605 10.1007/s11606-009-0945-5 19294472 PMC2669870

