# Supplementary material for: Geriatric assessment in undergraduate geriatric education – a structured interpretation guide improves the quantity and accuracy of the results: a cohort comparison
Source: BMC Med Educ. 2013 Aug 30;13:116. doi: 10.1186/1472-6920-13-116 (PMC3766262; doi:10.1186/1472-6920-13-116)
Supplement: Additional file 1 — Topics and content of the structured interpretation guide – an overview. [file 1472-6920-13-116-S1.doc]

**Additional file 1 -** Topics and content of the structured interpretation guide – an overview

| **topic** | **detailed content** |
| --- | --- |
| medication | - Are certain drugs omissible? - Are drug-drug interactions expectable? - Are specific side-effects associated with old age expectable? - Are important drugs in the patient’s medication missing or under-dosed? - Is the patient sufficiently familiar with his medication (purpose, taking)? - Does the patient take drugs the GP is not informed about? Do these drugs bring along dangerous side-effects or interactions? - Does the patient refuse to take drugs that have been prescribed by the GP? |
| physical fitness and mobility | - Is the patient limited in his mobility? For what reason? - Does the patient receive home visits? By whom? |
| general and domestic care | - Are the general and domestic care of the patient limited? - Is help available? - Provision of general and domestic help present? - Provision technical nursing present? - Are the patient’s relatives overextended? |
| (instrumental) activities of daily living (ADL/ IADL) | - Barthel-Index score: (0-60; 60-100) - Are the instrumental activities of daily living limited? - Is rehabilitation indicated? |
| social environment | - Is social support available? - Does the patient have financial problems? - Are architectonic problems present? - Does the patient receive any sort of service? |
| common geriatric cardinal symptoms | - Does the patient have dyspnoea? For what reason? - Does the patient have physical pain? - Does the patient have a sleeping disorder? - Did the patient involuntarily lose weight (or power)? - How many falls occurred? |
| physical status (medical history and examination) | - Is the BMI normal, too high or too low? - Does the patient have hypertension? - Does the patient have atrial fibrillation? - Does the patient have diabetes? - Did the patient experience a heart attack? - Did the patient experience a stroke? - Does the patient suffer from angina pectoris/ coronary heart disease? - Does the patient suffer from intermittent claudication? - Does the patient have dental problems? - Does the patient suffer from arthrosis? - Did the patient experience a bone fracture? - Does the patient suffer from incontinence? - Does the patient have difficulties with bowel movement? - Does the patient have a visual impairment? - Does the patient have a hearing impairment? |
| mood and cognition | - Are there signs for a depressive disorder? - Are there signs for dementia? Is the MMSE positive (<23 points)? |
| lifestyle | - Does the patient smoke? - Is the patient physically inactive? - Is the patient eating unhealthily? - Does the patient have problems with alcohol? |
| vaccination status | - Vaccination against influenza, pneumococci, and tetanus-diphtheria? |
| hospital admissions | - Has the patient been admitted to a hospital? |
| physical examination | - Is the patient’s blood pressure pathological? - Is the patient’s pulse regular? - Is the patient’s thorax symptomatic? - Is the patient’s strength lowered? - Is the patient’s flexibility symptomatic? - Is the patient’s foot status pathological? |
